# Potential Roles of Melatonin in Doxorubicin-Induced Cardiotoxicity: From Cellular Mechanisms to Clinical Application

**DOI:** 10.3390/pharmaceutics15030785

**Published:** 2023-02-27

**Authors:** Tanawat Attachaipanich, Siriporn C. Chattipakorn, Nipon Chattipakorn

**Affiliations:** 1 Cardiac Electrophysiology Research and Training Center, Faculty of Medicine, Chiang Mai University, Chiang Mai 50200, Thailand; 2 Center of Excellence in Cardiac Electrophysiology Research, Chiang Mai University, Chiang Mai 50200, Thailand; 3 Cardiac Electrophysiology Research Unit, Department of Physiology, Faculty of Medicine, Chiang Mai University, Chiang Mai 50200, Thailand

**Keywords:** melatonin, doxorubicin, anthracycline, cardiotoxicity, mitochondria

## Abstract

Doxorubicin is a potent chemotherapeutic drug; however, its clinical application has been limited due to its cardiotoxicity. One of the major mechanisms of doxorubicin-induced cardiotoxicity is the induction of oxidative stress. Evidence from in vitro and in vivo studies demonstrates that melatonin attenuated the increase in ROS production and lipid peroxidation from doxorubicin. Melatonin has been shown to exert protective effects on mitochondria damaged by doxorubicin via attenuating the depolarization of the mitochondrial membrane, restoring ATP production, and maintaining mitochondrial biogenesis. Doxorubicin increased mitochondrial fragmentation which impaired mitochondrial function; however, these adverse effects were reversed by melatonin. Melatonin also modulated cell death pathways by suppressing apoptotic and ferroptotic cell death caused by doxorubicin. These beneficial effects of melatonin could be responsible for the attenuation of changes in ECG, left ventricular dysfunction, and hemodynamic deterioration caused by doxorubicin. Despite these potential benefits, clinical evidence regarding the impact of melatonin in reducing cardiotoxicity induced by doxorubicin is still limited. Further clinical studies are justified to evaluate the efficacy of melatonin in protecting against doxorubicin-induced cardiotoxicity. This valuable information can be used to warrant the use of melatonin in a clinical setting under this condition.

## 1. Introduction

Doxorubicin, an anthracycline class chemotherapy drug, is a potent cancer treatment and is effective in treating various types of malignancies, including lymphoma, sarcoma, and breast cancer [1]. However, its clinical application is dose-limited due to the potential side effects of cancer therapy-related cardiac dysfunction (CTRCD) [2]. With the increased use of doxorubicin together with the advancement in cancer therapies and the increase in the rate of survival of cancer patients, doxorubicin-induced cardiotoxicity has raised serious concerns [2]. A prior retrospective study which included 4018 participants reported that the incidence of symptomatic heart failure from doxorubicin was 2.2% [3]. A later study that enrolled 630 breast and small cell lung cancer patients reported that the cumulative incidence of doxorubicin-related heart failure, including subclinical left ventricular (LV) dysfunction, was 5%, 26%, and 48% for the cumulative doses 400 mg/m^2^, 550 mg/m^2^, and 700 mg/m^2^, respectively [4]. Moreover, even a low dose of doxorubicin has been shown to increase the risk of LV dysfunction [4,5]. Results from a prospective study showed that patients who were treated with low to moderate doses of anthracycline, using doxorubicin equivalent doses ranging from 50 to 375 mg/m^2^, also subsequently developed subclinical LV dysfunction [5]. The risk of doxorubicin-related heart failure persisted for many years after chemotherapy completion, and the onset of symptoms could be delayed by up to two decades [1,6,7,8].

Current guidelines recommend the use of angiotensin-converting enzyme inhibitors (ACEIs) or angiotensin receptor blockers (ARBs) and beta-blockers for primary prevention in cancer patients at high risk for cardiovascular (CV) toxicity who are undergoing anthracycline chemotherapy [2,9]. A meta-analysis of small randomized trials demonstrated that ACEIs, ARBs, and beta-blockers effectively prevented a decline in LV ejection fraction (LVEF), but did not affect the incidence of clinical heart failure [10]. Additionally, dexrazoxane, an iron chelator, is also recommended for patients at high risk of CV toxicity who are receiving anthracycline therapy or those who are scheduled for high-dose anthracycline chemotherapy [2,9,11]. A recent meta-analysis of both randomized and non-randomized studies in breast cancer patients treated with anthracyclines showed that dexrazoxane reduces the risk of heart failure, although the quality of evidence remains limited [12].

There are multiple mechanisms involved in doxorubicin-induced cardiotoxicity. One of the major mechanisms is the increased oxidative stress by the overproduction of reactive oxygen species (ROS) [13]. Doxorubicin has been shown to become highly concentrated within mitochondria due to the strong affinity to cardiolipin, a negatively charged phospholipid component located at the inner mitochondrial membrane [14]. Since the cardiomyocytes have abundant mitochondria with a relatively low level of superoxide dismutase (SOD) and catalase (CAT) expression compared to other tissues, they were found to be vulnerable to doxorubicin-induced oxidative damage [15,16]. As a result, attempts to reduce the adverse effect of doxorubicin on the heart have been targeted on the reduction of oxidative stress [15].

Among many antioxidants available, melatonin, a potent antioxidant has been extensively investigated to prevent doxorubicin-induced cardiotoxicity [17]. Endogenous and exogenous melatonin were found to be highly concentrated within the mitochondria, which are the major source of ROS production and the targeted site of the doxorubicin [18,19]. Due to its amphiphilic property, melatonin was capable of crossing various biological membranes, including that of the mitochondria [18]. The molecular docking studies showed that the peptide transporters (PEPT) 1/2 and glucose transporter 1 (GLUT1) facilitated the uptake of melatonin and its accumulation within the mitochondria [20,21]. Melatonin not only exerted a protective effect against doxorubicin-induced cardiotoxicity via its direct antioxidative properties, but it also modulated the mitochondrial function and regulated the cell death pathways [22,23,24]. This review comprehensively summarizes the contemporary evidence from in vitro and in vivo studies together with clinical reports investigating the mechanisms regarding the potential protective effects of melatonin in doxorubicin-induced cardiotoxicity. Search criteria with the keywords melatonin, doxorubicin, anthracycline, chemotherapy, and cardiotoxicity were used to identify the relevant publications reported in the English language in the PubMed database from its inception to October 2022.

## 2. Effect of Melatonin on Oxidative Stress and Inflammatory Cytokines in Doxorubicin-Induced Cardiotoxicity: EVIDENCE from In Vitro and In Vivo Studies

Doxorubicin increased oxidative stress by generating ROS via a redox cycling mechanism [25]. Doxorubicin, a quinone compound, was reduced to a semiquinone, primarily by complex I of the electron transport chain (ETC) [25]. Subsequently, the semiquinone donated an unstable electron to an oxygen molecule and formed a superoxide anion (O_2_^−^) [25]. Normally, antioxidative enzymes defend against oxidative stress via the detoxification of the generated ROS. The SOD converted the superoxide anion to hydrogen peroxide (H_2_O_2_), which was then antioxidized by CAT or glutathione peroxidase enzyme (GPx) into a water molecule [26]. The hydrogen peroxide could then react with iron via the Fenton reaction and be converted to a potent hydroxyl radical (OH^•^) [26]. Doxorubicin also directly forms a complex with iron via a non-enzymatic reaction which results in oxidative damage [27].

Melatonin exerted antioxidative properties via direct scavenging of free radicals, both ROS and reactive nitrogen species (RNS), mainly by single electron transfer, hydrogen transfer, and radical adduction formation [28]. Due to its electron-rich molecule, melatonin was not found to undergo redox cycling like other antioxidants and did not promote oxidation. With these properties, melatonin acts as a terminal or suicidal antioxidant [29]. Melatonin was found to be converted to cyclic 3-hydroxymelatonin (3-OHM) after reactions with hydroxy free radicals. After oxidation, 3-OHM was then converted to N^1^-acetyl-N^2^-formyl-5-methoxykynuramine (AFMK), which subsequently deformylated to N^1^-acetyl-5-methoxykynuramine (AMK) [17]. These melatonin metabolites also exerted ROS scavenging capacity, and were considered as integral to a scavenging cascade reaction [29]. Melatonin also suppressed the expression of pro-oxidant enzymes, including nitric oxide synthase (NOS), and stimulated the expression of antioxidative enzymes including SOD, GPx, and glutathione reductase in various models, including those involving neuronal cells and hepatocytes [17,29]. In addition, melatonin modulated mitochondrial function by maintaining mitochondrial homeostasis, improving mitochondrial respiration, and stabilizing the mitochondrial membrane which attenuated the oxidative stress caused by doxorubicin [17].

In H9c2 cells, doxorubicin increased ROS production, which was attenuated with melatonin treatment [24,30,31,32]. The GPx activity was significantly decreased in H9c2 cells after being treated with doxorubicin, and melatonin pretreatment attenuated the reduction of the GPx activity [24]. Malondialdehyde (MDA) levels were increased in doxorubicin-treated H9c2 cells and isolated ventricular rat cardiomyocytes [30,33]. In these models, melatonin effectively attenuated the increase in lipid peroxidation caused by doxorubicin [30,33].

Consistent with in vitro reports, evidence from in vivo studies also demonstrated increased ROS production in rats and mice after being treated with doxorubicin [22,30]. Melatonin attenuated the increase in ROS production caused by doxorubicin [22,30]. Doxorubicin also increased nitric oxide (NO) levels in both rat and mouse models, however melatonin cotreatment effectively attenuated the increase in the NO level [34,35,36]. A reduced glutathione (GSH) level was also reduced in rats and mice treated with doxorubicin, melatonin therapy attenuating the reduction of GSH level [36,37,38,39,40,41]. Doxorubicin treatment reduced the activity of GPx in rats and mice, but melatonin pretreatment mitigated the decrease [24,30,42,43].

Regardless of these reports, some inconsistent findings exist. Two studies reported an increase in GPx activity after doxorubicin treatment [38,39], whereas melatonin treatment was shown to attenuate the increase in the GPx activity caused by doxorubicin [39]. Some studies even reported no effect after both doxorubicin and melatonin treatment on the GPx activity [35,38]. These discrepancies could be attributed to the different cumulative doses of doxorubicin, as well as the duration of cellular response to oxidative stress. Studies that reported a decrease in GPx activity following doxorubicin treatment used greater cumulative doses of doxorubicin and had longer intervals between doxorubicin treatment and time of enzymatic measurement [24,30,42,43].

It has been shown that acute exposure to doxorubicin caused a decrease in SOD activities in both rat and mouse models [30,34,35,42,43]. However, pretreatment with melatonin attenuated the decrease in SOD activity [30,42,43]. In contrast, chronic exposure to doxorubicin increased SOD activity, with several studies reporting no effect of melatonin on the enzyme [34,35,38]. In a rat model, acute exposure to doxorubicin reduced CAT activity, but melatonin pretreatment did not affect this [34]. Conversely, chronic exposure to doxorubicin significantly increased the CAT activity, with this activity being further increased by melatonin pretreatment [38]. The differences in cumulative dosage and treatment duration could be responsible for the discrepancies in the SOD and CAT activity induced by doxorubicin and melatonin treatments.

Doxorubicin significantly increased the level of lipid peroxidation products, including MDA, 4-hydroxyalkenals (4-HDA), thiobarbituric acid reactive substances (TBARS), conjugated dienes (CD), and protein carbonyl in both rat and mouse models. Melatonin cotreatment attenuated the doxorubicin-induced increase in lipid peroxidation [22,24,30,33,34,35,36,37,38,39,40,41,42,43,44,45,46,47]. Interestingly, using the pinealectomized rat model, higher MDA levels were observed when compared to the control group after the doxorubicin treatment [48]. Furthermore, an exogenous melatonin supplement further diminished lipid peroxidation in both groups [48]. These findings suggested that doxorubicin-induced oxidative damage could be prevented by melatonin in both physiologic and pharmacologic doses [48].

In a rat model, daunorubicin exposure that was both acute and subacute led to an increase in lipid peroxidation [38]. Treatment with melatonin markedly attenuated lipid peroxidation following subacute daunorubicin exposure [38]. There was no change in MDA and GSH levels after exposure to epirubicin and melatonin in rats [49]. Epirubicin elevated the NO level in cases in which it was attenuated by melatonin cotreatment [49]. In a rat model, trastuzumab, which has been used to treat breast cancer concurrently with doxorubicin, potentiated the doxorubicin-induced cardiotoxicity by increasing lipid peroxidation, reducing GPx, and reducing SOD activity [42]. It was demonstrated that these cardiotoxicities were abolished by melatonin cotreatment [42]. Some studies have reported that doxorubicin increased the level of inflammatory cytokines, including IL-1, IL-6, IL-18, and TNF-α [22,34,35]. These cytokines were markedly reduced after melatonin treatment [22,34,35].

All of these in vitro and in vivo findings indicated that melatonin exerted antioxidative effects by attenuating the increase in oxidative stress, lipid peroxidation, and inflammatory cytokines caused by doxorubicin. These effects of melatonin on oxidative stress and inflammatory cytokines reported from in vitro and in vivo studies are comprehensively summarized in Table 1.

## 3. Effects of Melatonin on Mitochondrial Functions, Biogenesis, and Dynamics in Doxorubicin-Induced Cardiotoxicity: Evidence from In Vitro and In Vivo Studies

### 3.1. Effect of Melatonin on Mitochondrial Functions in Doxorubicin-Induced Cardiotoxicity

Cardiolipin was essential for the optimal functions of complexes I, III, and IV of the ETC [51,52]. The interactions between doxorubicin and cardiolipin resulted in an inhibition of mitochondrial complex proteins, which subsequently impaired mitochondrial respiration, increased oxidative stress, and led to cell death [14]. The increase in mitochondrial ROS production due to doxorubicin caused mitochondrial damage through impaired mitochondrial bioenergetics, damaged mitochondrial DNA (mt-DNA), decreased mitochondrial membrane potential (MMP), and increased mitochondrial permeability transition pore (mPTP) opening, all of which promoted cellular apoptosis [53].

Doxorubicin was shown to reduce MMP, ATP production, and mt-DNA content in neonatal rat cardiomyocytes and H9c2 cells [23,24,30,31,32,45]. Treatment with melatonin alleviated the doxorubicin-induced decrease in MMP, ATP production, and mt-DNA content [23,24,30,31,32,45]. A study in H9c2 cells showed that doxorubicin decreased the expression of complexes I-IV, which were attenuated with melatonin cotreatment [32]. Moreover, melatonin has been reported to enhance the mitochondrial respiratory chain activity in other tissues. The results from studies in the brain and liver mitochondria from rats and mice showed that melatonin increased the activity of complexes I, III, and IV [54,55].

Consistent with in vitro reports, in vivo studies in rat and mouse models also demonstrated that doxorubicin increased mitochondrial swelling along with lowering MMP, ATP production, and mt-DNA content [22,24]. Melatonin attenuated these mitochondrial changes resulting from the doxorubicin treatment in these models [22,24]. Doxorubicin decreased the expression of complex I in rats, and melatonin cotreatment normalized the level of expression of complex I from the doxorubicin [22]. Studies in rat and mouse models revealed that doxorubicin decreased stage 3 respiration, increased stage 4 respiration, and lowered the respiratory control ratio between stages 3 and 4 [22,56,57,58,59,60]. Melatonin cotreatment potentially prevented the decrease of the respiratory control ratio after doxorubicin treatment [22].

The protective effects of melatonin on mitochondria were also demonstrated in various models. Evidence from an in vivo study showed that melatonin protected against mitochondrial uncoupling from ruthenium red by attenuating the decrease of the activity of mitochondrial complexes I and IV [18]. Melatonin increased complex IV activity along with LV systolic function in a sepsis-induced cardiomyopathy rat model [61].

Yes-associated protein (YAP), a downstream effector of the Hippo signaling pathway, is essential in various physiologic and pathologic processes [62,63]. Evidence from in vitro studies in H9c2 cells and mouse cardiomyocytes showed that YAP promoted the transcription of antioxidative enzymes, including CAT and SOD, which provided protective effects in a cardiac I/R injury model [62]. Doxorubicin diminished YAP expression along with its downstream targeted genes, including CTGF, Birc5, and Park2 in H9c2 cells and mouse models [31]. Melatonin pretreatment mitigated the effect of doxorubicin on the reduction of YAP and its downstream target gene expression [31]. In H9c2 cells and in rats, doxorubicin also inhibited YAP activation and increased phosphorylated YAP (p-YAP), and treatment with melatonin restored the p-YAP/YAP level [24]. In models of YAP downregulation using si-RNA knockout in H9c2 cells and verteporfin-treated rats, it has been demonstrated that the antioxidative effect and mitochondrial protection exerted by melatonin were abolished in the case of induced doxorubicin cardiotoxicity [24,31]. After YAP was downregulated, the beneficial effects of melatonin on attenuating ROS production, decreasing lipid peroxidation, and protecting the mitochondria including attenuating MMP decline, ATP depletion, and reduced mt-DNA content from doxorubicin, were reversed [24,31]. These results suggested that melatonin exerted the antioxidative effects and preserved YAP in the mitochondria.

All of these findings suggested that melatonin protected mitochondria against doxorubicin by enhancing the mitochondrial respiratory complex activity, attenuating the decrease in MMP, and restoring the decrease in the mt-DNA content. These reports are comprehensively summarized in Table 2.

### 3.2. Effect of Melatonin on Mitochondrial Biogenesis in Doxorubicin-Induced Cardiotoxicity

Peroxisome proliferator-activated receptor-γ coactivator-1α (PGC-1α) was the main regulator of mitochondrial biogenesis, a process which involved the expression of multiple metabolic enzymes and mitochondrial respiratory complexes [64]. The PGC-1α activity was enhanced by AMP-activated protein kinase (AMPK) and Sirtuin 1 (SIRT1) via posttranslational phosphorylation and deacetylation, respectively [64]. The active PGC-1α stimulated multiple downstream transcriptional factors, including the nuclear respiratory factor (NRF) which regulated the expression of the mitochondrial respiratory chain proteins and mitochondrial antioxidative enzymes, mitochondrial transcription factor A (TFAM), which was responsible for the replication and transcription of the mt-DNA, and uncoupling protein 2 (UCP2), which reduced oxidative stress [15,64,65].

Evidence from an in vitro study showed that doxorubicin significantly decreased the expression of PGC-1α and its downstream signaling compounds which included NRF1, TFAM, and UCP2 in H9c2 cells [30]. Moreover, phosphorylated AMPK (p-AMPK) was decreased after treatment with doxorubicin. Melatonin treatment attenuated the decrease of PGC-1α, NRF1, UCP2, TFAM, and p-AMPK [30]. Knockdown of AMPK or PGC-1α in H9c2 cells partially reversed the protective effects of melatonin on a decrease in ROS production, and lipid peroxidation, and increased ATP production from the doxorubicin [30].

AMPK was also found to be an important regulator of cellular homeostasis and mitochondrial biogenesis [66]. AMPK is composed of three subunits, including the catalytic domain (α subunit) and 2 regulatory domains (β, γ subunits) [66,67]. There were two different AMPKα isoforms, including AMPKα1, which is ubiquitously expressed, and AMPKα2, which is mainly expressed in tissues with a high metabolism, such as cardiomyocytes [66,67]. In the mouse embryonic fibroblasts (MEFs), doxorubicin was shown to decrease AMPKα1 expression in those cells [32]. Moreover, the AMPKα1-deficient MEFs had significantly higher apoptotic rates after being treated with doxorubicin, compared to the wild type, indicating the pro-survival effect of AMPKα1 [68]. Conversely, doxorubicin increased the expression of AMPKα2 at the transcriptional level via transcription factor E2F1 and induced apoptosis in H9c2 cells and MEFs [32,69]. The overexpression of AMPKα2 in H9c2 cells potentiated the effect of doxorubicin with regard to increasing ROS production, decreasing MMP, impairing ATP production, reducing mt-DNA content, and inducing cellular apoptosis [32]. Consistently, AMPKα2-deficient MEFs showed the attenuation of those adverse effects caused by the doxorubicin [32]. Interestingly, evidence from H9c2 cells, MEFs, and mouse models demonstrated that melatonin attenuated an increase in transcription factor E2F1 activity, an increase in both cellular and mitochondrial AMPKα2 expression, and a decrease of the AMPKα1 expression caused by doxorubicin, which could be responsible for the protective effects of melatonin [32].

The SIRT1, an NAD^+^-dependent histone, deacetylase, mediated the survival of cardiomyocytes and protected against apoptosis in various stress conditions [70]. Evidence from studies in H9c2 cells, neonatal rat cardiomyocytes, and mice showed that doxorubicin downregulated the expression of SIRT1 [70,71,72]. A study in a tumor-bearing rat model showed that doxorubicin decreased SIRT1 expression, and melatonin cotreatment attenuated the reduction of SIRT1 expression in this model [23]. Thus, melatonin could exert a protective effect via SIRT1 preservation.

All of these findings indicated that melatonin maintained mitochondrial biogenesis by attenuating the decrease of PGC-1α, together with its downstream and upstream signaling in doxorubicin-induced cardiotoxicity models. Moreover, melatonin preserved mitochondrial function and attenuated cell death, which had resulted from doxorubicin through the upregulation of SIRT1 expression and downregulation of AMPKα2. These reports are comprehensively summarized in Table 2.

### 3.3. Effect of Melatonin on Mitochondrial Dynamics in Doxorubicin-Induced Cardiotoxicity

Mitochondria are dynamic organelles and their morphologies can shift between isolated organelles and extensive networks in response to alterations in signaling cascades and stress conditions, which is essential for cell survival and function [73]. The fusion process of mitochondria involves the activity of mitofusin-1 (Mfn1) and mitofusin-2 (Mfn2), which are located at the outer mitochondrial membrane, and optic atrophy 1 (OPA1), which is located at the inner mitochondrial membrane [73]. Mitochondrial fission involves the activity of dynamin-related protein1 (Drp1) and human mitochondrial fission 1 protein (hFis1) [73]. Doxorubicin-induced cardiotoxicity could be the result of an imbalance in mitochondrial dynamics by suppressing mitochondrial fusion and promoting mitochondrial fission [15]. Doxorubicin has been shown to enhance mitochondrial fission, resulting in mitochondrial fragmentation which impaired mitochondrial function, increased oxidative stress, and subsequently led to cellular apoptosis [15,74].

Evidence from an in vitro study showed that doxorubicin decreased OPA1 and increased the FUN14 domain containing 1 (FUNDC1), and melatonin pretreatment attenuated these effects in H9c2 cells [24]. Previous in vivo studies reported consistent findings that doxorubicin decreased the expression of mitochondrial fusion proteins, including Mfn1, Mfn2, and OPA1, and increased mitochondrial fission proteins, including Drp1, phosphorylated Drp1, hFis1, and FUNDC1, in rats [22,23,24]. Melatonin attenuated the decrease in mitochondrial fusion proteins and the increase in mitochondrial fission proteins, resulting from the doxorubicin [22,23,24]. The histologic examination also revealed that melatonin decreased mitochondrial fragmentation from the doxorubicin [23,32]. YAP downregulation was shown to abrogate the beneficial effects of melatonin on the impairment of mitochondrial dynamic balance resulting from doxorubicin treatment [24].

All of these findings indicated that melatonin attenuated the increase in mitochondrial fission and restored the impairment of mitochondrial fusion proteins caused by doxorubicin. Treatment with melatonin restored mitochondrial dynamic balance, resulting in the maintenance of mitochondrial function, reducing oxidative stress, and attenuating cell death. The effects of melatonin on mitochondrial functions, biogenesis, and dynamics from in vitro and in vivo studies are comprehensively summarized in Table 2.

## 4. Effect of Melatonin on Cellular Death Pathways in Doxorubicin-Induced Cardiotoxicity: Evidence from In Vitro and In Vivo Studies

### 4.1. Effect of Melatonin on Apoptosis in Doxorubicin-Induced Cardiotoxicity

Doxorubicin triggered cardiomyocyte cell death via multiple regulated cell death pathways [75]. Apoptosis has been the most extensively studied and was one of the major regulated cell death mechanisms responsible for the doxorubicin-induced cardiotoxicity [75,76]. Doxorubicin increased the opening of mPTP and induced mitochondrial calcium accumulation, which induced MMP disruption, and cytochrome c release, and led to apoptotic cell death [77]. The oxidative stress from doxorubicin also enhanced apoptosis since the cardiolipin, which was sensitive to oxidative stress, became peroxidized and subsequently induced mPTP opening and the release of cytochrome c [15,78].

Melatonin potentially attenuated apoptotic cell death from doxorubicin by inducing antioxidative effects and maintaining mitochondrial function [77]. Previous studies showed that the mPTP inhibitor, cyclosporine, could attenuate the doxorubicin-induced cardiotoxicity in isolated rat hearts and human atrial cardiomyocytes [79,80].

Evidence from in vitro and in vivo studies supported the antiapoptotic role of melatonin in protecting against doxorubicin-induced cell death. Previous in vitro studies showed that doxorubicin increased the pro-apoptotic protein, Bcl-2 associated X protein (Bax), and decreased the anti-apoptotic protein, Bcl-2, in H9c2 cell and zebrafish models [24,30,31,32]. The rate of apoptosis and hence apoptotic markers including cleaved caspase (c-caspase) 3, c-caspase 9, and cleaved poly (ADP-ribose) polymerase-1 (c-PARP-1) were also increased after being treated with doxorubicin [24,30,31,32]. Melatonin attenuated the increase in Bax/Bcl-2 and decreased the apoptotic rate and apoptotic markers in H9c2 cells, zebrafish, and mouse fibroblast cells [24,30,31,32,81].

Previous in vivo studies also reported enhanced apoptosis, increased Bax, and decreased Bcl-2 in rats and mice after being treated with the doxorubicin [22,23,24,30,82]. Melatonin effectively attenuated the increase in the rate of apoptosis, and the levels of apoptotic markers and Bax/Bcl-2 caused by doxorubicin [22,23,24,30,82].

The down regulation of YAP in H9c2 cells and in rats showed that the anti-apoptotic effects of melatonin against doxorubicin-induced apoptotic cell death were diminished [24,31]. This result suggested that the anti-apoptotic effect of melatonin against doxorubicin-induced cardiotoxicity was partly through YAP activation [24,31].

All of these findings indicated that melatonin prevented doxorubicin-induced cellular apoptosis by exerting antioxidative effects, inhibiting the opening of mPTP, attenuating the increase in pro-apoptotic proteins, and decreasing anti-apoptotic proteins, which occur as a consequence of doxorubicin treatment. These reports are comprehensively summarized in Table 3.

### 4.2. Effect of Melatonin on Autophagy and Mitophagy in Doxorubicin-Induced Cardiotoxicity

Autophagy is a degradative cellular process using lysosomes that prevent cellular damage, promote cell survival, and maintain cellular function in response to various stimuli [83]. The results from earlier studies on the effect of doxorubicin on autophagy were found to be conflicting [84]. Previous in vitro and in vivo studies in neonatal rat cardiomyocyte, H9c2 cell, and rat models reported that doxorubicin enhanced autophagy [85,86,87,88]. However, several studies using H9c2 cells and in mice showed that doxorubicin decreased autophagy [89,90]. This discrepancy could be due to the use of different models, variations in the cumulative doses of doxorubicin, and intervals from doxorubicin exposure to autophagic measurement [75,84]. Recent evidence from studies in neonatal rat ventricular cardiomyocyte and mouse models suggested that doxorubicin enhanced the early steps of autophagy, but inhibited the later steps by disrupting lysosomal acidification which leads to the accumulation of undegraded autolysosomes [91,92]. This accumulation of dysfunctional autolysosomes enhances oxidative damage and cell death [91,92]. The inhibition of the early steps of autophagy could reduce the accumulation of autolysosomes and ROS production from the doxorubicin [91]. In rats, it has been shown that doxorubicin increased Beclin-1, p62, and microtubule-associated protein 1A/1B-light chain 3 (LC3)-II/LC3-I. Interestingly, cotreatment with melatonin effectively attenuated the increase in autophagy caused by doxorubicin [22].

Damaged mitochondria could be specifically removed by selective autophagy, known as mitophagy [83]. After a mitochondrial damage insult, PTEN-induced kinase 1 (Pink1) and Parkin were found to be involved in the labeling of damaged mitochondria for the process of mitophagy to occur [83]. Evidence from in vitro and ex vivo studies in AC16 cells and Langendorff perfused heart models showed that doxorubicin enhanced mitophagy, and treatment with the mitophagy inhibitor, mdivi-1, exerted protective effects against doxorubicin by attenuating the decrease of MMP and PGC-1α, along with associated downstream signaling [93,94]. However, there was evidence that indicated that there was an inhibitory effect on mitophagy via the Pink/parkin pathway by doxorubicin [95]. The discrepancy found with regard to the Pink/parkin pathway associated mitophagy could be explained by the different models of doxorubicin treatment and the time frame of mitochondrial damage due to the biphasic change of the Pink/parkin level after mitochondrial damage [96,97].

An ROS scavenger was involved in mitophagy regulation, as evidenced by the attenuation of the increase in mitophagy and the protective effects caused by the mitochondrial ROS scavenger mito-tempo in AC16 cells treated with doxorubicin [94]. Melatonin, as a mitochondrial ROS scavenger, could potentially also decrease mitophagy and protect against doxorubicin. Evidence from an in vivo study showed that doxorubicin increased Pink1 and Parkin levels in rats, and, although melatonin pretreatment normalized the Pink1 level, it did not affect the Parkin level [23]. However, another study in rats reported no significant change in Pink1 and Parkin after being treated with doxorubicin and melatonin [22].

All of these findings suggested that melatonin attenuated the increase in autophagy and might inhibit the mitophagy which result from doxorubicin treatment. A comprehensive summary of these reports is shown in Table 3.

### 4.3. Effect of Melatonin on Ferroptosis in Doxorubicin-Induced Cardiotoxicity

Ferroptosis is a form of a regulated cell death pathway which is characterized by iron-dependent lipid peroxidation, and could be prevented by iron chelation [98,99]. Evidence from neonatal rats, mice, and the cardiomyocytes from doxorubicin-treated patients showed that doxorubicin increased the accumulation of mitochondrial iron [100]. The study models, which showed the attenuation of the accumulation of iron in the mitochondria, including one in transgenic mice overexpressing the mitochondrial iron exporting protein ABC protein-B8 (ABCB8) and another in cardiomyocytes of neonatal rat treated with dexrazoxane, illustrated protective effects of melatonin against the doxorubicin-induced cardiotoxicity [100]. Ferroptosis was one of the major cell death pathways in the doxorubicin-induced cardiotoxicity, and ferroptosis inhibition could exert cardioprotective effects [76,101]. Doxorubicin-induced cell death was partially prevented by using ferrostatin-1 (a ferroptosis inhibitor) [76]. Ferrostatin-1 increased the survival rate of mice treated with a single dose of doxorubicin, whereas emricasan (an apoptosis inhibitor), necrostatin-1 (a necroptosis inhibitor), and 3-methyladenine (3-MA, an autophagy inhibitor), had no effect on survival [101]. Melatonin has been shown to exert an antiferroptotic effect in various models [102,103,104].

An in vitro study in H9c2 cells and an in vivo study in rat models demonstrated that doxorubicin increased the level of Acyl-CoA synthase long-chain family member 4 (ACSL4) and decreased glutathione peroxidase 4 (GPx4), which then promoted ferroptosis [24]. Melatonin attenuated these doxorubicin-induced effects [24]. Cardiomyocytes from mice treated with doxorubicin displayed histologic changes consistent with the process of ferroptosis, including mitochondrial swelling, the loss of cristae, and the rupture of the outer mitochondrial membrane [24]. The melatonin therapy reversed these changes in the mitochondrial ultrastructure [24]. Interestingly, YAP downregulation in both H9c2 cells and rat models reversed the antiferroptotic effect of melatonin on attenuating the increase in ACSL4 and ferroptosis-like mitochondrial morphological changes from the doxorubicin [24]. These results suggested that the antiferroptotic activity of melatonin was partly via the preservation of the YAP expression impaired by doxorubicin [24].

**Figure 1:**
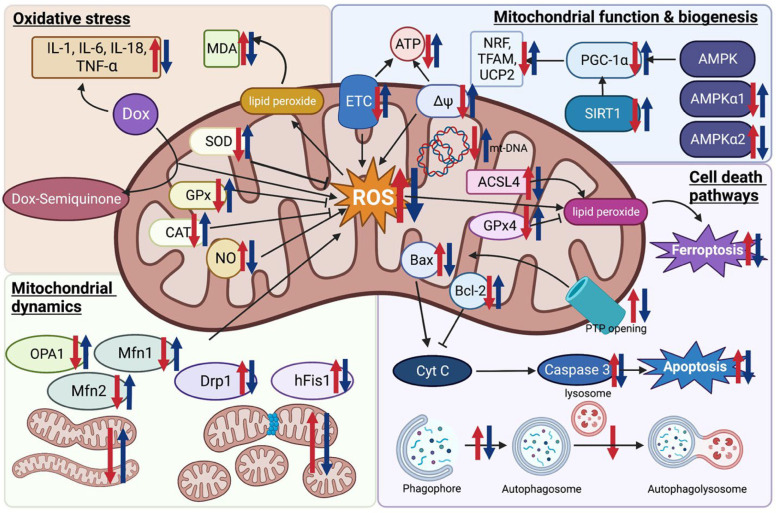
Potential effects of melatonin on oxidative stress, mitochondrial function, biogenesis, and dynamics, and cell death pathways in doxorubicin-induced cardiotoxicity. Red arrows indicate the changes occurring in a doxorubicin model compared to the control. Blue arrows indicate the changes after being treated with melatonin in comparison to the doxorubicin model. Figure created with BioRender.com (accessed on 7 January 2023). ACSL4 Acyl-CoA synthase long-chain family member 4; AMPK, AMP-activated protein kinase; Bax, Bcl-2 associated X protein; CAT, catalase; Cyt C, cytochrome c; Dox, doxorubicin; Drp1, dynamin-related protein1; ETC, electron transport chain; GPx, glutathione peroxidase; hFis1, human mitochondrial fission 1 protein; MDA, malondialdehyde; Mfn, mitofusin; mPTP, mitochondrial permeability transition pore; mt-DNA, mitochondrial DNA; NO, nitric oxide; NRF, nuclear respiratory factor; OPA1, optic atrophy 1; PGC-1α, peroxisome proliferator-activated receptor-γ coactivator-1α; ROS, reactive oxygen species; SIRT1, Sirtuin 1; SOD, superoxide dismutase; TFAM, mitochondrial transcription factor A; UCP2, uncoupling protein 2; ∆Ψ mitochondrial membrane potential.

All of these findings indicated that melatonin exerted cardioprotection against doxorubicin-induced cardiotoxicity which occurred via ferroptosis inhibition. The effect of melatonin on apoptosis, autophagy, mitophagy, and ferroptosis from in vitro and in vivo studies are comprehensively summarized in Table 3. The potential mechanisms conferring the benefits of melatonin in doxorubicin-induced cardiotoxicity are illustrated in Figure 1.

## 5. Effect of Melatonin on ECG, LV Function, and Hemodynamics in Doxorubicin-Induced Cardiotoxicity: Evidence from In Vivo and Ex Vivo Studies

In addition to molecular mechanisms, functional evidence, including ECG, LV function, and hemodynamic changes, are also key factors determining the potential clinical benefits of melatonin against doxorubicin-induced cardiotoxicity. Various ECG parameters were reported as being affected by doxorubicin exposure. In rats, doxorubicin increased both QT and corrected QT (QTc) intervals, and pretreatment with melatonin effectively attenuated these changes [24,34,35,105]. However, one study reported that melatonin had no effect on the QT and QTc prolongation caused by doxorubicin [34]. Doxorubicin also increased the amplitude of the ST-segment elevation, and pretreatment with melatonin attenuated this change [43,105]. One study also reported that doxorubicin increased ST-segment depression, and melatonin pretreatment reversed this change [24]. The PR interval was also prolonged in rats treated with doxorubicin; however, melatonin did not affect this parameter [34,35]. Doxorubicin was also shown to reduce the duration of the QRS complex, whereas melatonin pretreatment prevented this change caused by doxorubicin [43,105]. However, one study did report that doxorubicin increased the duration of the QRS complex, whereas melatonin cotreatment had no impact on this [34]. Although there were some discrepancies in the effects of doxorubicin and melatonin on ECG parameters, most studies demonstrated that melatonin had beneficial effects on the attenuation of ECG changes caused by doxorubicin. These discrepancies in ECG changes could be due to the use of different animal models, pharmacologic treatment protocols, and intervals between medication and ECG assessment. Future studies are needed to verify this issue.

Doxorubicin has been linked to cardiac arrhythmias in both acute and chronic settings, potentially due to its impact on abnormal automaticity, trigger activities, and reentrant circuits [106]. The short-term exposure of doxorubicin has been associated with a decrease in connexin-43 expression in mice, even after a single dose of doxorubicin [107]. This reduction of connexin-43 expression has resulted in abnormal electrical conduction and APD dispersion, which facilitates the arrhythmogenesis [108]. Melatonin has been shown to possess anti-arrhythmic properties, preventing ventricular fibrillation inducibility in ex vivo study using an old guinea pig heart model [109]. In addition, melatonin has been found to enhance connexin-43 expression in a spontaneous hypertensive rat model, leading to a reduction in susceptibility to arrhythmias [110]. Melatonin has also been shown to mitigate the decrease in connexin-43 expression, its lateralization, cardiac fibrosis, and arrhythmic susceptibility in catecholamine overdrive in normotensive and hypertensive rat models [111]. Furthermore, melatonin has been found to reduced ventricular fibrillation in obesity rat models by attenuating the decrease in connexin-43 expressions and its lateralization [112]. Oxidative stress has been identified as a potential mechanism of arrhythmogenesis in doxorubicin-treated patients [113]. Treatment with antioxidants, such as vitamin E and N-acetylcysteine, has been shown to attenuate the APD prolongation in rats treated with doxorubicin [114]. As a potent antioxidant, melatonin may also provide an anti-arrhythmic effect in this setting. Further studies on the anti-arrhythmic effect of melatonin on doxorubicin-induced cardiotoxicity are essentially needed to fully understand its mechanism and potential clinical applications.

With regard to the aspect of echocardiographic assessment, LV systolic function parameters, including LVEF and fractional shortening (FS) were impaired after being treated with doxorubicin in rat and mouse models [22,24,30,31,44,50]. LV diastolic function parameters, including early to late diastolic transmitral flow velocity (E/A), were also impaired following doxorubicin treatment [22]. Melatonin attenuated the decrease of the LVEF, FS, and E/A from the doxorubicin [22,24,30,31,44,50]. Doxorubicin also increased LV end-diastolic pressure (LVEDP), and melatonin cotreatment effectively attenuated this change [22,33,45,82].

Doxorubicin impaired other cardiac function parameters in rat and mouse models, including the treatment showing a correlation with a decrease in stroke volume (SV), dP/dt_max_, dP/dt_min_, and cardiac output (CO), but melatonin attenuated these changes [22,23,33,82]. Doxorubicin was reported to reduce heart rate (HR), and melatonin treatment attenuated this decrease [22,24,31,33,43,45,82]. However, some studies reported no change in HR from the doxorubicin and melatonin treatment [23,82]. In rat models, doxorubicin reduced systolic blood pressure (SBP), diastolic blood pressure (DBP), and mean arterial blood pressure (MAP) but melatonin effectively restored those parameters in these in vivo models [22,44,45].

Heart rate variability (HRV) was a noninvasive test that reflected the activity of the sympathetic and vagal components of the autonomic nervous system [115]. Previous studies assessing the effect of doxorubicin on HRV showed that the drug increased the low frequency (LF)/high frequency (HF) ratio which indicated cardiac sympathetic overactivity or parasympathetic withdrawal [22,116]. Melatonin cotreatment attenuated the increase in LF/HF resulting from the doxorubicin [22].

All of these findings indicated that melatonin attenuated the ECG changes from doxorubicin, including QT prolongation and ST-segment amplitude. Both LV systolic and diastolic dysfunction from doxorubicin were improved with melatonin cotreatment. Melatonin also improved the SV, CO, HR, and BP changes resulting from doxorubicin. The effect of melatonin on ECG, LV function, and hemodynamics from in vivo and ex vivo studies are comprehensively summarized in Table 4.

## 6. Effect of Melatonin on Tumor Cells and Future Clinical Perspectives

Due to the antioxidative and antiapoptotic effects of melatonin, the potential interference in the efficacy of doxorubicin cancer treatment is a major concern. Evidence from various in vitro and in vivo studies demonstrated that melatonin had dual effects on apoptosis by selectively modulating the antiapoptotic effect on normal cells, and triggering the apoptotic pathways in cancer cells [117]. Treatment with melatonin alone has the potential to reduce cell proliferation and enhance apoptosis in many cancer cell lines, including breast cancer, those involving hematologic malignancies, and prostate cancer [117,118,119,120,121]. Studies in various cancer models have been extensively reported regarding the mechanisms behind melatonin’s anticancer effects, including the induction of tumor cell apoptosis and the reduction of cell proliferation though the upregulation of p53, increased Bax, and decreased Bcl-2 in gastric, renal, and breast cancer cells, as well as ovarian cancer rats models [122,123,124,125,126]. Melatonin also increased the expressions of Bim, a pro-apoptotic Bcl-2 protein, in liver and renal cancer cells [127,128]. Additionally, melatonin has been found to increase oxidative stress in colorectal cancer cells and to have an anti-angiogenesis effect by downregulating VEGF in breast and liver cancer cells [129,130,131]. Moreover, melatonin has a potential to decrease tumor invasiveness by suppressing expressions of matrix metallopeptidase (MMP) 2 and 9, which play a key role in the degradation of the extracellular matrix, in breast and liver cancer cell lines [132,133].

Melatonin also synergized the anti-tumor effects with doxorubicin in breast cancer cell lines and tumor-bearing rat models [22,23,134]. A randomized study in advanced-stage solid tumor patients reported the efficacy of melatonin in enhancing the action of the chemotherapy on tumor regression [135]. A small study in advanced-stage cancer patients reported that the subgroup of patients without tumor progression had a decline in VEGF levels after being treated with melatonin [136]. A randomized study on metastatic colorectal cancer patients who did not respond to 5-FU treatment revealed that melatonin combined with low-dose IL-2 improved 1-year survival, compared to supportive treatment alone [137]. Further, a randomized study on advanced solid organ tumors showed that melatonin treatment increased patients’ survival rate compared to supportive treatment alone [138]. Additionally, a randomized study in advanced solid organ tumors found that melatonin supplementation reduced chemotherapy toxicity, including neurotoxicity, cytopenia, and cachexia, and improved the survival rate and tumor regression [139]. A randomized study in advanced stage breast cancer also showed that the combination of melatonin and tamoxifen improved tumor response compared to tamoxifen alone [140]. These studies supported the potential role of melatonin as a preventive strategy in doxorubicin-induced cardiotoxicity without interfering with the cancer treatment effects of doxorubicin.

The research on the combination of anthracycline chemotherapy and radiotherapy is still limited. Evidence suggests that melatonin enhances the radiotherapy effect on cancer cells. Previous in vitro studies have indicated that melatonin improves the radiotherapy effect on breast cancer cells through the modulation of p53 and DNA break repaired proteins [141,142]. A small randomized study in glioblastoma patients found that melatonin treatment dose 20 mg in conjunction with radiotherapy at 60 Gy significantly improved 1-year survival and reduced radiotherapy-associated toxicity compared to the radiotherapy alone group [143]. On the other hand, a study of brain metastasis patients who underwent whole-brain radiotherapy reported no benefit of melatonin dose 20 mg on survival [144].

Melatonin has also been shown to prevent the radiotherapy effect on normal cells. In vitro and in vivo studies have demonstrated that melatonin has a radioprotective effect in various cell types, including lymphocytes, germ cells, and mice exposed to whole-body irradiations [145]. Clinical studies have indicated that melatonin can mitigate the potential side effects of radiotherapy, such as alopecia and oral mucositis [143,146]. However, the protective effect of melatonin on cardiotoxicity from radiotherapy remains uncertain due to limited evidence. In vivo studies in rats showed that pretreatment with 50 mg/kg of melatonin IP before radiation 18 Gy can prevent fibrosis, vasculitis, and myocardial necrosis [147]. Another study in xenograft mice with colon cancer found that pretreatment with melatonin 20 mg/kg IP prior to radiotherapy 5 Gy reduced oxidative damage to cardiac tissue. Overall, the evidence suggests that the protective effect of melatonin on doxorubicin-induced cardiotoxicity would persist with concurrent radiotherapy treatment.

Although extensive evidence from both in vitro and in vivo studies demonstrated the effectiveness of melatonin in attenuating doxorubicin-induced cardiotoxicity, there are still limited clinical studies. A randomized study in 250 advanced-stage solid tumor patients with poor functional status reported that melatonin potentially improved the 1-year survival rate and reduced the incidence of cardiotoxicity [135]. Further clinical studies are warranted, and indeed to be recommended to further evaluate the use of melatonin as a preventative strategy for ameliorating the effects of the cardiotoxicity cause by doxorubicin.

## 7. Conclusions

Evidence from both in vitro and in vivo studies demonstrates the potential benefits of melatonin on doxorubicin-induced cardiotoxicity. Melatonin exerted antioxidative properties, had mitochondrial protective effects, and prevented cell death from doxorubicin by suppressing apoptosis, attenuating autophagy dysregulation, and decreasing ferroptosis. The ECG change and LV dysfunction from doxorubicin was also attenuated with melatonin. Melatonin has the potential to exert anti-arrhythmic properties against doxorubicin, but further studies are necessary to fully comprehend its underlying mechanisms and assess its clinical potential. Melatonin provided protection for cardiomyocytes without interfering with the efficacy of cancer treatment from doxorubicin. Overall, melatonin could be a potential candidate for a therapeutic strategy in the prevention of doxorubicin-induced cardiotoxicity. However, further high-quality clinical studies are still required to validate the efficacy of melatonin for future clinical application.

## Figures and Tables

**Table 1:** Effects of melatonin on oxidative stress and inflammatory cytokines in doxorubicin-induced cardiotoxicity: evidence from in vitro and in vivo studies.

Model	Drug/Dose/Route/Duration/Condition	Parameters			Interpretation	Ref.
		Lipid Peroxidation	Oxidative Stress and Antioxidative Enzyme	Inflammatory Cytokine		
In vitro studies						
Isolated ventricular rat cardiomyocytes	Dox/300 µM/4 h	↑↑MDA	-	-	Mel attenuated the increase in lipid peroxidation from Dox-induced toxicity.	[33]
	Dox + Mel/1 or 3 µM/4 h/cotreat	↑MDA	-	-		
H9c2 cells	Dox/1 µM/24 h	-	↑↑ROS	-	Mel attenuated oxidative stress caused by Dox via YAP activation.	[31]
	Dox + Mel/10 µM/24 h/pretreat 24 h	-	↑ROS	-		
H9c2 cells + si-RNA knockout YAP	Dox + Mel/10 µM/24 h/pretreat 24 h	-	↑↑ROS	-		
H9c2 cells	Dox/1 µM/24 h	-	↑↑ROS, ↓↓GPx	-	Mel attenuated the increase in ROS and the reduction in GPx caused by Dox via YAP activation.	[24]
	Dox + Mel/10 µM/24 h/pretreat 24 h	-	↑ROS, ↓GPx	-		
H9c2 cells + si-RNA knockout YAP	Dox + Mel/10 µM/24 h/pretreat 24 h	-	↑↑ROS	-		
H9c2 cells	Dox/1 µM/24 h	-	↑↑ROS	-	Mel attenuated the increase in ROS caused by Dox via reducing AMPKα2.	[32]
	Dox + Mel/1 mM/24 h/cotreat	-	↑ROS	-		
	Dox + NAC/5 mM	-	↑ROS	-		
	Dox + Mel + NAC	-	↔ROS	-		
H9c2 cells transfected with AMPKα2 WT	Dox/1 µM/24 h	-	↑↑↑ROS	-		
	Dox + Mel/1 mM/24 h/cotreat	-	↑↑ROS	-		
H9c2 cells transfected with AMPKα2 DN	Dox/1 µM/24 h	-	↑↑ROS	-		
	Dox + Mel/1 mM/24 h/cotreat	-	↑ROS	-		
Double knockout (α1^−/−^α2^−/−^) MEFs	Dox/1 µM/24 h	-	↑↑ROS	-		
	Dox + Mel/1 mM/24 h/cotreat	-	↑ROS	-		
*Ampk*α2^−/−^ MEFs	Dox/1 µM/24 h	-	↑↑ROS	-		
	Dox + Mel/1 mM/24 h/cotreat	-	↑ROS	-		
*Ampk*α1^−/−^ MEFs	Dox/1 µM/24 h	-	↑↑↑ROS	-		
	Dox + Mel/1 mM/24 h/cotreat	-	↑↑ROS	-		
*Ampk*α WT MEFs	Dox/1 µM/24 h	-	↑↑↑ROS	-		
	Dox + Mel/1 mM/24 h/cotreat	-	↑↑ROS	-		
H9c2 cells	Dox/1 µM/24 h	↑↑↑MDA	↑↑↑ROS	-	Mel attenuated the increase in ROS and lipid peroxidation caused by Dox via AMPK and PGC-1α activation.	[30]
	Dox + Mel/100 µM/24 h/cotreat	↑MDA	↑ROS	-		
H9c2 cells + si-RNA knockdown AMPK	Dox + Mel/100 µM/24 h/cotreat	↑↑MDA	↑↑ROS	-		
H9c2 cells + si-RNA knockdown PGC-1α	Dox + Mel/100 µM/24 h/cotreat	↑↑MDA	↑↑ROS	-		
In vivo studies						
Male Sprague-Dawley rats	Dox/15 mg/kg/IP/1 dose	↑MDA	-	-	Mel reversed the increase in lipid peroxidation from Dox.	[46]
	Dox + Mel/10 MKD/sc/5 days/pretreat 2 days	↔MDA	-	-		
Male Sprague-Dawley rats	Dox/15 or 25 mg/kg/IP/1 dose	↑MDA	-	-	Mel reversed the increase in lipid peroxidation from Dox.	[33]
	Dox + Mel/10 MKD/sc/6 days/pretreat 2 days	↔MDA	-	-		
Male Wistar rats	Dox/3 MKD/IP/6 doses	↑MDA	↑ROS	↑TNF-α, ↑IL-6	Mel attenuated the increase in lipid peroxidation, ROS, and inflammatory cytokines from Dox.	[22]
	Dox + Mel/10 MKD/PO/30 days/cotreat	↔MDA	↔ROS	↔TNF-α, ↔IL-6		
Non-Px Female Wistar rats (sham)	Dox/20 mg/kg/IP/1 dose	↑MDA	-	-	Both physiologic and pharmacologic doses of Mel attenuated the increase in lipid peroxidation from Dox.	[48]
	Dox + Mel/4 MKD/2 days/pretreat 1 h	↔MDA	-	-		
	Dox + Mel/4 MKD/2 days/posttreat 24 h	↔MDA	-	-		
Pinealectomized (Px) Female Wistar rats	Compared to sham	↑MDA	-	-		
	Dox/20 mg/kg/IP/1 dose	↑↑MDA	-	-		
	Dox + Mel/4 MKD/2 days/posttreat 24 h	↔MDA	-	-		
Male Buffalo strain rats	Dox/10 mg/kg/IV/1 dose	↑MDA+4-HDA	-	-	Mel attenuated the increase in lipid peroxidation from Dox.	[38]
	Dox + Mel/10 mg/kg/sc/pre and posttreat	↔MDA+4-HDA	-	-		
	Dox/3 mg/kg/wk/IV/x 3 wks	↑MDA+4-HDA	-	-		
	Dox + Mel/10 mg/kg/sc/pre and posttreat	↑MDA+4-HDA	-	-		
Male Sprague-Dawley rats	Dox/3 mg/kg/IV q 3 days/4 doses	↑↑TBARS, ↑CD	-	-	Mel attenuated the increase in lipid peroxidation from Dox.	[45]
	Dox + Mel/6 MKD/15 days/pretreat 1 day	↑TBARS, ↔CD	-	-		
Male Sprague-Dawley rats	Dox/2.5 mg/kg/IP/6 doses	↑TBARS	-	-	Mel attenuated the increase in lipid peroxidation from Dox.	[44,50]
	Dox + Mel/4 mg/kg/IP/3 wks/pretreat 1 wk	↔TBARS	-	-		
Male Wistar rats	Dox/45 mg/kg/IV/1 dose	↑↑MDA	↓GSH	-	Mel attenuated the increase in lipid peroxidation and a decrease of GSH from Dox.	[37]
	Dox + Mel/10 MKD/sc/7 days/pretreat 7 days	↑MDA	↔GSH	-		
	Dox + Mel/10 MKD/sc/7 days/posttreat	↔MDA	↑GSH	-		
Swiss albino mice bearing Ehrlich ascites carcinoma	Dox 4 mg/kg/wk/IP/4 doses	↑MDA	↓GSH, ↔SOD	-	Mel attenuated the increase in lipid peroxidation and a decrease of anti-oxidative enzyme from Dox.	[40]
	Dox + Mel 5 MKD/PO/30 days/pretreat 24 h	↔MDA	↔GSH, ↑SOD	-		
Male Swiss albino mice	Dox 2.5 MKD/IP/5 days	↑↑MDA	↓↓GSH, ↑↑NO	-	Mel attenuated the increase in lipid peroxidation, RNS and a decrease of GSH from Dox.	[36]
	Dox + Mel 1 MKD/IP/5 days/pretreat 30 mins	↑↑MDA	↓↓GSH, ↑↑NO	-		
	Dox + Mel 5 MKD/IP/5 days/pretreat 30 mins	↑MDA	↓GSH, ↑NO	-		
Male Wistar rats	Dox 20 mg/kg/IP/1 dose	↑MDA + 4-HDA	↓GSH, ↓GPx	-	Mel normalized the increase in lipid peroxidation and a decrease of GSH from Dox.	[41]
	Dox + Mel 50 µg/kg/day/IP/10 days/pretreat 4 days	↔MDA + 4-HDA	↔GSH, ↓GPx	-		
Male Sprague-Dawley rats	Dox 15 mg/kg/IP/1 dose	↑↑MDA	↓↓SOD, ↓↓CAT, ↓↓GPx, ↑↑NO	-	Mel attenuated the increase in lipid peroxidation, RNS and a decrease of anti-oxidative enzyme from Dox.	[47]
	Dox + Mel 5 MKD/IP/10 days/pretreat 3 days	↑MDA	↓SOD, ↓CAT, ↓GPx, ↑NO			
Male Wistar Albino rats	Dox/45 mg/kg/IV/1 dose	↑MDA	↓SOD, ↓CAT, ↔GPx, ↑NO, ↑HIF-1α, ↓TAS, ↑TOS	↑IL-1, ↑IL-6, ↑IL-18, ↑TNF-α	Mel attenuated the increase in lipid peroxidation, RNS, and inflammatory cytokines from Dox.	[34]
	Dox + Mel/10 MKD/IP/7 days/pretreat 4 days	↔MDA	↓SOD, ↓CAT, ↔GPx ↔NO, ↑HIF-1α, ↓TAS, ↑TOS	↔IL-1, ↔IL-6, ↔IL-18, ↔TNF-α		
Male Wistar albino rats	Dox/45 MKD/IV/1 dose	↑MDA	↔NO, ↔HIF-1α, ↔TAS, ↔TOS, ↔GPx, ↔CAT, ↓SOD	↑IL-1, ↑IL-6, ↑IL-18, ↔TNF-α	Mel reversed the increase in lipid peroxidation and inflammatory cytokines from Dox.	[35]
	Dox + Mel/10 MKD/IP/7 days/pretreat 4 days	↔MDA	↓NO, ↔HIF-1α, ↔TAS, ↔TOS, ↔GPx, ↔CAT, ↓SOD	↔IL-1, ↔IL-6, ↓IL-18, ↔TNF-α		
Male Wistar-albino rats	Dox/18 mg/kg/IP/3 days	↑MDA	↓SOD, ↓ GPx	-	Mel attenuated the increase in lipid peroxidation and a decrease in anti-oxidative enzymes from Dox.	[43]
	Dox + Mel/10 MKD/IP/7 days/pretreat 4 days	↔MDA	↑SOD, ↔GPx	-		
Male C57BL/6 mice	Dox/10 mg/kg/IP/2 days	↑↑↑MDA	↑↑↑ROS, ↓↓↓SOD, ↓↓↓GPx	-	Mel attenuated the increase in lipid peroxidation, ROS and decrease of anti-oxidative enzymes from Dox via AMPK activation.	[30]
	Dox + Mel/20 MKD/IP/8 days/pretreat 1 day	↑MDA	↑ROS, ↓SOD, ↓GPx	-		
	Dox + Mel + selective AMPK inhibitor	↑↑MDA	↑↑ROS, ↓↓SOD, ↓↓GPx	-		
Male Sprague Dawley rats	Dox/5 mg/kg/wk/5 wks	↑MDA	↓GPx	-	Mel reversed the increase in lipid peroxidation and decrease in GPx from Dox via YAP activation.	[24]
	Dox + Mel/10 mg/kg/wk/IP/5 wks/pretreat 24 h	↔MDA	↔GPx	-		
	Dox + Mel + Verteporfin 1 mg/100g/wk/IP/pre Mel 5 wks	↑MDA	↔ GPx	-		
Male Sprague Dawley rats	Dox/10 mg/kg/IP/1 dose	↑↑TBARS, ↑↑protein carbonyl	↓GSH, ↓GST, ↑↑GPx, ↑CAT	-	Mel attenuated the increase in lipid peroxidation and decrease of GSH from Dox.	[39]
	Dox + Mel/15 MKD/IP/10 days/pretreat 5 days	↑TBARS, ↑protein carbonyl	↔GSH, ↔GST, ↑GPx, ↔CAT	-		
Buffalo strain rats	Dox/2.5 mg/kg/wk/IP/4 wks	↑↑MDA + 4-HDA	↑GPx, ↓↓GSH, ↑SOD, ↑CAT	-	Mel attenuated the increase in lipid peroxidation and a decrease of GSH from Dox.	[38]
	Dox + Mel/20 mg/kg/wk/sc/4 wks/pretreat 15 min	↑MDA + 4-HDA	↑GPx, ↔GSH, ↑SOD, ↑↑CAT	-		
Male Buffalo strain rats	Dau/10 mg/kg/IV/1 dose	↑MDA + 4-HDA	-	-	Mel attenuated the increase in lipid peroxidation from Dau.	[38]
	Dau + Mel/10 mg/kg/sc/pre and posttreat	↑MDA + 4-HDA	-	-		
	Dau/3 mg/kg/wk/IV/x 3 wks	↑MDA + 4-HDA	-	-		
	Dau + Mel/10 mg/kg/sc/pre and posttreat	↔MDA + 4-HDA	-	-		
Male Wistar rats	Epi/10 mg/kg/IP/1 dose	↔MDA	↑NO, ↔GSH	-	Mel normalized the increase in RNS from Epi.	[49]
	Epi + Mel/200 µg/kg/IP/10 days/cotreat	↔MDA	↔NO, ↔GSH	-		
Male Sprague-Dawley rats	Dox/20 mg/kg/IP/1 dose	↑↑MDA	↓↓GPx, ↓↓SOD	-	Mel attenuated the increase in lipid peroxidation and a decrease in anti-oxidative enzymes from Dox and Trast.	[42]
	Dox + Mel/10 mg/kg/PO bid/pretreat 36 h and posttreat 72 h	↑MDA	↓GPx, ↓SOD	-		
	Dox + Trast/10 mg/kg/IP/1 dose	↑↑↑MDA	↓↓↓GPx, ↓↓↓SOD	-		
	Dox + Mel + Trast	↑MDA	↓ GPx, ↓SOD	-		

-Not applicable; AMPK, AMP-activated protein kinase; CAT, catalase, CD, conjugated dienes; Dau, daunorubicin; DN, double negative; Dox, doxorubicin; Epi, Epirubicin; GPx, glutathione peroxidase; GSH, reduced glutathione; HIF-1α, hypoxia-inducible factor 1-α; IP, intraperitoneal; MDA, malondialdehyde; MEFs, mouse embryonic fibroblasts; Mel, melatonin; NAC, N-acetyl cysteine; NO, nitric oxide; PGC-1α, peroxisome proliferator-activated receptor-γ coactivator-1α; ROS, reactive oxygen species; RNS, reactive nitrogen species; SOD, superoxide dismutase; TAS, total anti-oxidative stress; TBARS, thiobarbituric acid reactive substances; Trast, trastuzumab; TOS, total oxidative stress; WT, wild-type; YAP, Yes-associated protein; 4-HAD, 4-hydroxyalkenals. Each arrow symbol represents a comparison to the control group: ↔ indicates no significant change compared to the control group. ↑, ↑↑, ↑↑↑ indicate a significant increase compared to the control group. ↑↑ indicates a further increase compared to the previous condition in the same model, which showed a ↑ compared to the control group. ↑↑↑ indicates a further increase compared to the condition in the same model, which showed a ↑↑ compared to the control group. ↓, ↓↓, ↓↓↓ indicate a significant decrease compared to the control group. ↓↓ indicates a further decrease compared to the previous condition in the same model, which showed a ↓ compared to the control group. ↓↓↓ indicates a further decrease compared to the condition in the same model, which showed a ↓↓ compared to the control group.

**Table 2:** Effects of melatonin on mitochondrial structure, function, biogenesis, and dynamics in doxorubicin-induced cardiotoxicity: evidence from in vitro and in vivo studies.

Model	Drug/Dose/Route/Duration	Parameters			Interpretation	Ref.
		Structure and Function	Biogenesis	Dynamics		
In vitro studies						
Neonatal rat cardiomyocytes	Dox/20 µM/24 h	↓MMP	-	-	Mel attenuated the decrease of MMP from Dox.	[45]
	Dox + Mel/1 mmol/L/pretreat 1 h	↔MMP	-	-		
H9c2 cells	Dox/1 µM/24 h	↓↓MMP	-	-	Mel reversed the decrease of MMP from Dox via YAP activation	[31]
	Dox + Mel/10 µM/24 h/pretreat 24 h	↔MMP	-	-		
H9c2 cells + si-RNA knockout YAP	Dox + Mel/10 µM/24 h/pretreat 24 h	↓MMP	-	-		
H9c2 cells	Dox/1 µM/24 h	↓↓MMP, ↓↓ATP, ↓↓mtDNA	↑↑AMPKα2, ↑p-AMPKα2, ↓complex I-V	-	Mel attenuated the decrease of MMP, ATP, and mt-DNA from Dox via reducing AMPKα2	[32]
	Dox + Mel/1 mM/24 h/cotreat	↓MMP, ↓ATP, ↓mtDNA	↑AMPKα2, ↔p-AMPKα2	-		
H9c2 cells transfected with AMPKα2 WT	Dox/1 µM/24 h	↓↓↓MMP, ↓↓↓ATP, ↓↓↓mtDNA	-	-		
	Dox + Mel/1 mM/24 h/cotreat	↓↓MMP, ↓ATP, ↓mtDNA	-	-		
H9c2 cells transfected with AMPKα2 DN	Dox/1 µM/24 h	↓↓MMP, ↓↓ATP, ↓↓mtDNA	-	-		
	Dox + Mel/1 mM/24 h/cotreat	↓MMP, ↓ATP, ↓mtDNA	-	-		
Double knockout (α1^−/−^α2^−/−^) MEFs	Dox/1 µM/24 h	↓↓ATP, ↓↓MMP, ↓mito length	-	-		
	Dox + Mel/1 mM/24 h/cotreat	↓ATP, ↓MMP, ↓mito length	-	-		
*Ampk*α2^−/−^ MEFs	Dox/1 µM/24 h	↓↓ATP, ↓↓MMP, ↓mito length	↓↓AMPKα1	-		
	Dox + Mel/1 mM/24 h/cotreat	↓ATP, ↓MMP, ↓mito length	↓AMPKα1	-		
*Ampk*α1^−/−^ MEFs	Dox/1 µM/24 h	↓↓↓ATP, ↓↓↓MMP, ↓↓mito length	↑↑AMPKα2	-		
	Dox + Mel/1 mM/24 h/cotreat	↓ATP, ↓↓MMP, ↓mito length	↑AMPKα2	-		
*Ampk*α WT MEFs	Dox/1 µM/24 h	↓↓↓ATP, ↓↓↓MMP, ↓↓mito length	↓AMPKα1, ↑↑AMPKα2	-		
	Dox + Mel/1 mM/24 h/cotreat	↓ATP, ↓↓MMP, ↓mito length	↓AMPKα1, ↑AMPKα2	-		
H9c2 cells	Dox/1 µM/24 h	↓↓↓ATP	↓↓PGC-1α, ↓↓↓NRF1, ↓↓↓UCP2, ↓↓↓TFAM, ↓↓p-AMPK	-	Mel attenuated the decrease of ATP production resulting from Dox via AMPK and PGC-1α activation.	[30]
	Dox + Mel/100 µM/24 h/cotreat	↓ATP	↓PGC-1α, ↓NRF1, ↓UCP2, ↓TFAM, ↓p-AMPK	-		
H9c2 cells + si-RNA knockdown AMPK	Dox + Mel/100 µM/24 h/cotreat	↓↓ATP	↓↓PGC-1α, ↓↓NRF1, ↓↓UCP2, ↓↓TFAM, ↓↓↓p-AMPK	-		
H9c2 cells + si-RNA knockdown PGC-1α	Dox + Mel/100 µM/24 h/cotreat	↓↓ATP	↓↓↓PGC-1α, ↓↓↓NRF1, ↓↓↓UCP2, ↓↓↓TFAM, ↓p-AMPK	-		
H9c2 cells	Dox/3 µM/24 h	↓ATP, ↓mito major/minor aspect, ↓mito branching	-	-	Mel attenuated the decrease of ATP production and mitochondrial fragmentation, resulting from Dox.	[23]
	Dox + Mel/10 µM/24 h/pretreat 24 h	↔ATP, ↔mito major/minor aspect, ↔mito branching	-	-		
H9c2 cells	Dox/1 µM/24 h	↓↓MMP, ↓↓ATP, ↓↓mtDNA	-	↑↑FUNDC1, ↓↓OPA1	Mel attenuated the decrease of MMP, ATP, mt-DNA, and mitochondrial fusion from Dox via YAP activation.	[24]
	Dox + Mel/10 µM/pretreat 24 h	↓MMP, ↓ATP, ↓↓mtDNA	-	↑FUNDC1, ↓OPA1		
H9c2 cells +si-RNA knockout YAP	Dox + Mel/10 µM/pretreat 24 h	↓↓MMP	-	↑↑FUNDC1, ↓↓OPA1		
In vivo studies						
Male Wistar rats	Dox/3 MKD/IP/6 doses	↓↓MMP ↑↑swelling	↓PGC-1α ↓complex I ↔complex II-V	↓Mfn-1, ↓↓Mfn-2, ↓↓OPA1, ↑p-Drp1	Mel attenuated the decrease of MMP, complex I of ETC, and mitochondrial fusion and an increase in mitochondrial fission from Dox.	[22]
	Dox + Mel/10 MKD/PO/30 days/cotreat	↓MMP ↑swelling	↔PGC-1α, ↔complex I-V	↔Mfn-1, ↓Mfn-2, ↓OPA1, ↔p-Drp1		
Male C57BL/6 mice	Dox/10 mg/kg/IP/2 days	-	↓↓PGC-1α, ↓↓↓NRF1, ↓↓↓UCP2, ↓↓↓TFAM, ↓↓p-AMPK	-	Mel attenuated the decrease of PGC-1α and its downstream signaling via AMPK activation.	[30]
	Dox + Mel/20 MKD/IP/8 days/pretreat 1 day	-	↓PGC-1α, ↓NRF1, ↓UCP2, ↓TFAM, ↓p-AMPK	-		
	Dox + Mel + selective AMPK inhibitor	-	↓↓PGC-1α, ↓↓NRF1, ↓↓UCP2, ↓↓TFAM, ↓↓p-AMPK	-		
Female Sprague Dawley rats inoculates with LA7 rat mammary adenocarcinoma tumor cell	Dox/4 mg/kg/IP/3 doses	-	↓PGC-1α, ↓SIRT1	↔Mfn-1, ↓Mfn-2, ↑Drp1, ↑hFis1	Mel attenuated the decrease of PGC-1α, SIRT1, and mitochondrial fusion, and an increase in mitochondrial fission from Dox.	[23]
	Dox + Mel/6 MKD/PO/14 days/pretreat 3 days	-	↑PGC-1α, ↔SIRT1	↔Mfn-1, ↔Mfn-2, ↔Drp1, ↔hFis1		
Male Sprague Dawley rats	Dox/5 mg/kg/wk/IP/5 wks	↓↓ATP, ↓↓mtDNA, ↑swelling	-	↑↑FUNDC1, ↓↓OPA1	Mel attenuated the decrease of ATP, mt-DNA, and mitochondrial fusion from Dox via YAP activation.	[24]
	Dox + Mel/10 mg/kg/wk/IP/5 wks/pretreat 24 h	↓ATP, ↓mtDNA, ↔swelling	-	↑FUNDC1, ↓OPA1		
	Dox + Mel + Verteporfin 1 mg/100 g/wk/IP/pre Mel 5 wks	↓↓ATP, ↓↓mtDNA, ↑swelling	-	↑↑FUNDC1, ↓↓OPA1		

-Not applicable; AMPK, AMP-activated protein kinase; DN, double negative; Dox, doxorubicin; Drp1, dynamin-related protein1; ETC, electron transport chain; FUNDC1, FUN14 domain containing 1; hFis1, human mitochondrial fission 1 protein; IP, intraperitoneal; MEFs, mouse embryonic fibroblasts; Mel, melatonin; Mfn, mitofusin; mito, mitochondria; mtDNA, mitochondrial DNA; MKD, mg/kg/day; NRF, nuclear respiratory factor; OPA1, optic atrophy 1; PGC-1α, peroxisome proliferator-activated receptor-γ coactivator-1α; SIRT1, Sirtuin 1; TFAM, mitochondrial transcription factor A; UCP2, uncoupling protein 2; WT, wild-type; YAP, Yes-associated protein. Each arrow symbol represents a comparison to the control group: ↔ indicates no significant change compared to the control group. ↑, ↑↑, ↑↑↑ indicate a significant increase compared to the control group. ↑↑ indicates a further increase compared to the previous condition in the same model, which showed a ↑ compared to the control group. ↑↑↑ indicates a further increase compared to the condition in the same model, which showed a ↑↑ compared to the control group. ↓, ↓↓, ↓↓↓ indicate a significant decrease compared to the control group. ↓↓ indicates a further decrease compared to the previous condition in the same model, which showed a ↓ compared to the control group. ↓↓↓ indicates a further decrease compared to the condition in the same model, which showed a ↓↓ compared to the control group.

**Table 3:** Effects of melatonin on cellular death pathways in doxorubicin-induced cardiotoxicity: evidence from in vitro and in vivo studies.

Model	Drug/Dose/Route/Duration	Parameters			Interpretation	Ref.
		Apoptosis	Autophagy and Mitophagy	Ferroptosis		
In vitro studies						
H9c2 cells	Dox/1 µM/24 h	↑↑Bax, ↓↓Bcl-2, ↑↑↑apoptosis	-	-	Mel attenuated the increase in apoptosis from Dox via YAP activation.	[31]
	Dox + Mel/10 µM/24 h/pretreat 24 h	↑Bax, ↓Bcl-2, ↑apoptosis	-	-		
H9c2 cells +si-RNA knockout YAP	Dox + Mel/10 µM/24 h/pretreat 24 h	↑↑Bax, ↓↓Bcl-2, ↑↑apoptosis	-	-		
H9c2 cells	Dox/1 µM/24 h	↑↑↑apoptosis, ↑↑↑c-PARP1, ↑↑↑c-caspase3	-	-	Mel attenuated the increase in apoptosis from Dox via reducing AMPKα2.	[32]
	Dox + Mel/1 mM/24 h/cotreat	↑↑apoptosis, ↑c-PARP1, ↑c-caspase3	-	-		
	Dox + NAC/5 mM	↑↑apoptosis	-	-		
	Dox + Mel + NAC/5 mM	↑apoptosis	-	-		
	Dox + Mel + 4-P-PDOT/10 µM	↑↑c-PARP1, ↑↑c-caspase3	-	-		
	Dox + Mel + 4-P-PDOT/20 µM	↑↑↑c-PARP1, ↑↑↑c-caspase3	-	-		
H9c2 cells transfected with AMPKα2 WT	Dox/1 µM/24 h	↑↑↑↑apoptosis	-	-		
	Dox + Mel/1 mM/24 h/cotreat	↑↑↑apoptosis	-	-		
H9c2 cells transfected with AMPKα2 DN	Dox/1 µM/24 h	↑↑apoptosis	-	-		
	Dox + Mel/1 mM/24 h/cotreat	↑apoptosis	-	-		
Double knockout (α1^−/−^α2^−/−^) MEFs	Dox/1 µM/24 h	↑↑apoptosis	-	-		
	Dox + Mel/1 mM/24 h/cotreat	↑apoptosis	-	-		
*Ampk*α2^−/−^ MEFs	Dox/1 µM/24 h	↑↑apoptosis	-	-		
	Dox + Mel/1 mM/24 h/cotreat	↑apoptosis	-	-		
*Ampk*α1^−/−^ MEFs	Dox/1 µM/24 h	↑↑↑apoptosis	-	-		
	Dox + Mel/1 mM/24 h/cotreat	↑↑apoptosis	-	-		
*Ampk*α WT MEFs	Dox/1 µM/24 h	↑↑↑apoptosis	-	-		
	Dox + Mel/1 mM/24 h/cotreat	↑↑apoptosis	-	-		
NIH3T3 cells	Dox/2.6 µM/24 h	↑↑apoptosis	-	-	Mel attenuated the increase in apoptosis from Dox.	[81]
	Dox + Mel/1 µM/24 h/cotreat	↑apoptosis	-	-		
H9c2 cells	Dox/1 µM/24 h	↑↑↑Bax, ↓↓↓Bcl2, ↑↑↑c-caspase3, ↑↑↑apoptosis	-	-	Mel attenuated the increase in apoptosis from Dox via AMPK and PGC-1α activation.	[30]
	Dox + Mel/100 µM/24 h/cotreat	↑Bax, ↓Bcl2, ↑c-caspase3, ↑apoptosis	-	-		
H9c2 cells + si-RNA knockdown AMPK	Dox/1 µM/24 h + Mel/100 µM/24 h/cotreat	↑↑Bax, ↓↓Bcl2, ↑↑c-caspase3	-	-		
H9c2 cells + si-RNA knockdown PGC-1α	Dox/1 µM/24 h + Mel/100 µM/24 h/cotreat	↑↑apoptosis	-	-		
zebrafish	Dox 15 µM/120 h	↑↑BAX/Bcl-2, ↑c-caspase9, ↓↓caspase9	-	-	Mel attenuated the increase in apoptosis from Dox.	[24]
	Dox + Mel/50 µM/120 h/cotreat	↑BAX/Bcl-2, ↔c-caspase9, ↓caspase9	-	-		
H9c2 cells	Dox/1 µM/24 h	↑BAX/Bcl-2, ↑c-caspase9, ↓caspase9	-	↑ACSL4, ↓↓GPX4	Mel attenuated the increase in apoptosis and ferroptosis from Dox via YAP activation.	[24]
	Dox + Mel/10 µM/24 h/pretreat 24 h	↔BAX/Bcl-2, ↔c-caspase9, ↔caspase9	-	↔ACSL4, ↓GPX4		
H9c2 cells +si-RNA knockout YAP	Dox/1 µM/24 h + Mel/10 µM/24 h/pretreat 24 h	↑BAX/Bcl-2, ↑c-caspase9, ↓caspase9	-	↑ACSL4, ↓GPX4		
In vivo studies						
Male Wistar rats	Dox/3 MKD/IP/6 doses	↑↑Bax/Bcl2, ↑↑c-caspase3, ↑↑apoptosis	↔PINK1, ↔Parkin, ↑Beclin-1, ↑p62, ↑LC3-II/LC3-I	-	Mel attenuated the increase in apoptosis and autophagy from Dox.	[22]
	Dox + Mel/10 MKD/PO/30 days/cotreat	↑Bax/Bcl2, ↑c-caspase3, ↑apoptosis	↔PINK1, ↔Parkin, ↔Beclin-1, ↔p62, ↔LC3-II/LC3-I	-		
Male C57BL/6 mice	Dox/10 mg/kg/IP/2 days	↑↑↑Bax, ↓↓↓Bcl2, ↑↑↑c-caspase3, ↑↑↑apoptosis	-	-	Mel attenuated the increase in apoptosis from Dox via AMPK activation.	[30]
	Dox + Mel/20 MKD/IP/8 days/pretreat 1 day	↑Bax, ↓Bcl2, ↑c-caspase3, ↑apoptosis	-	-		
	Dox + Mel + selective AMPK inhibitor	↑↑Bax, ↓↓Bcl2, ↑↑c-caspase3, ↑↑apoptosis	-	-		
Female Sprague Dawley rats inoculated with LA7 rat mammary adenocarcinoma tumor cells	Dox/4 mg/kg/IP/3 doses	↑c-PARP, ↑c-caspase3	↑↑Pink1, ↑Parkin	-	Mel attenuated the increase in apoptosis and Pink1 from Dox.	[23]
	Dox + Mel/6 MKD/PO/14 days/pretreat 3 days	↔c-PARP, ↔c-caspase3	↔Pink1, ↑Parkin	-		
ICR mice	Dox 22.5 mg/kg/IP/1 dose	↑↑apoptosis	-	-	Mel attenuated the increase in apoptosis from Dox.	[82]
	Dox + Mel/2.5 µg/hr/sc/5 days/pretreat 24 h	↑apoptosis	-	-		
Male Sprague Dawley rats	Dox/5 mg/kg/wk/IP/5 wks	↑BAX/Bcl-2, ↑c-caspase9, ↓caspase9	-	↑ACSL4, ↓↓GPX4	Mel attenuated the increase in apoptosis and ferroptosis from Dox via YAP activation.	[24]
	Dox + Mel/10 mg/kg/wk/IP/5 wks/pretreat 24 h	↔BAX/Bcl-2, ↔c-caspase9, ↔caspase9	-	↔ACSL4, ↓GPX4		
	Dox + Mel + Verteporfin 1 mg/100 g/wk/IP/pre Mel 5 wks	↑BAX/Bcl-2, ↑c-caspase9, ↓caspase9	-	↑ACSL4, ↓GPX4		

- Not applicable; ACSL4, acyl-CoA synthase long-chain family member 4; AMPK, AMP-activated protein kinase; Bax, Bcl-2 associated X protein; c-caspase, cleaved caspase; DN, double negative; Dox, doxorubicin; GPx, glutathione peroxidase; IP, intraperitoneal; LC3, microtubule-associated protein 1A/1B-light chain 3; Mel, melatonin; PARP1, poly (ADP-ribose) polymerase-1; Pink1, PTEN-induced kinase 1; WT, wild-type; YAP, Yes-associated protein. Each arrow symbol represents a comparison to the control group: ↔ indicates no significant change compared to the control group. ↑, ↑↑, ↑↑↑ indicate a significant increase compared to the control group. ↑↑ indicates a further increase compared to the previous condition in the same model, which showed a ↑ compared to the control group. ↑↑↑ indicates a further increase compared to the condition in the same model, which showed a ↑↑ compared to the control group. ↓, ↓↓, ↓↓↓ indicate a significant decrease compared to the control group. ↓↓ indicates a further decrease compared to the previous condition in the same model, which showed a ↓ compared to the control group. ↓↓↓ indicates a further decrease compared to the condition in the same model, which showed a ↓↓ compared to the control group.

**Table 4:** Effects of melatonin on ECG, LV function, and hemodynamics in doxorubicin-induced cardiotoxicity: evidence from in vivo and ex vivo studies.

Model	Drug/Dose/Route/Duration	Parameters				Interpretation	Ref.
		ECG	LVEF	E/A	Hemodynamics		
In vivo studies							
Male Wistar rats	Dox/3 MKD/IP/6 doses	-	↓↓	↓↓	↓SBP, ↓DBP, ↓HR, ↓↓SV, ↑↑LF/HF	Mel attenuated the LV systolic and diastolic dysfunction from Dox.	[22]
	Dox + Mel/10 MKD/PO/30 days/cotreat	-	↓	↓	↔SBP, ↔DBP, ↔HR, ↓SV, ↑LF/HF		
C57BL/6 mice	Dox/5 mg/kg/wk/IP/5 wks	-	↓↓	-	↓↓HR	Mel attenuated the LV systolic dysfunction and a decrease of HR from Dox.	[31]
	Dox + Mel/10 mg/kg/IP/pretreat 24 h	-	↓	-	↓HR		
Male C57BL/6 mice	Dox/10 mg/kg/IP/2 days	-	↓↓↓	-	-	Mel attenuated the LV systolic dysfunction from AMPK activation.	[30]
	Dox + Mel/20 MKD/IP/8 days/pretreat 1 day	-	↓	-	-		
	Dox + Mel + selective AMPK inhibitor	-	↓↓	-	-		
Male Wistar rats	Dox/18 MKD/IP/3 days	↑STE, ↑QT, ↓P, ↓QRS	-	-	-	Mel normalized the ECG changes from Dox.	[105]
	Dox + Mel/40 MKD/IP/7 days/pretreat 4 days	↔STE, ↔QT, ↔P, ↔QRS	-	-	-		
Male Sprague-Dawley rats	Dox/3 mg/kg/IV q 3 days/4 doses	-	-	-	↓SBP, ↓DBP, ↓MAP, ↓HR	Mel attenuated the decrease in blood pressure and HR from Dox.	[45]
	Dox + Mel/6 MKD/IP/15 days/pretreat 1 day	-	-	-	↔SBP, ↔DBP, ↔MAP, ↔HR		
Female Sprague Dawley rats inoculated with LA7 rat mammary adenocarcinoma tumor cell	Dox/4 mg/kg/IP/3 doses	-	-	-	↓CO, ↓SV, ↔HR	Mel attenuated the decrease in CO and SV from Dox.	[23]
	Dox + Mel/6 MKD/PO/14 days/pretreat 3 days	-	-	-	↔CO, ↔SV, ↔HR		
Male Wistar albino rats	Dox/45 MKD/IV/1 dose	↑PR, ↑QRS, ↑QT, ↑↑QTc, ↑RR	-	-	-	Mel attenuated the QTc prolongation from Dox.	[35]
	Dox + Mel/10 MKD/IP/7 days/pretreat 4 days	↑PR, ↑QRS, ↑QT, ↑QTc, ↑↑RR	-	-	-		
Male Wistar-albino rats	Dox/18 mg/kg/IP/3 days	↑STE, ↓R, ↑P, ↓QRS, ↔QT, ↑RR	-	-	↑HR	Mel attenuated the ECG changes from Dox.	[43]
	Dox + Mel/10 MKD/IP/7 days/pretreat 4 days	↔STE, ↓R, ↓P, ↔QRS, ↔QT, ↔RR	-	-	↔HR		
ICR mice	Dox 22.5 mg/kg/IP/1 dose	-	-	-	↔HR, ↓SV, ↓CO	Mel attenuated the decrease in CO and SV from Dox.	[82]
	Dox + Mel/2.5 µg/hr/sc/5 days/pretreat 24 h	-	-	-	↔HR, ↔SV, ↔CO		
Male Sprague Dawley rats	Dox/5 mg/kg/wk/IP/5 wks	↑↑QT, ↑↑RR, ↓↓ST	↓	-	-	Mel attenuated the LV systolic dysfunction from Dox via YAP activation.	[24]
	Dox + Mel/10 mg/kg/wk/IP/5 wks/pretreat 24 h	↑QT, ↑RR, ↓ST	↔	-	-		
	Dox + Mel + Verteporfin 1 mg/100 g/wk/IP/pre Mel 5 wks	↑↑QT, ↑↑RR, ↓↓ST	↓	-	-		
Ex vivo study							
ICR mice	Dox/5 µM/for 60 min	-	-	-	↓HR	Mel attenuated the decrease of HR from Dox.	[82]
	Dox + Mel/1 µM/60 min/pretreat 5 min	-	-	-	↔HR		

-Not applicable; AMPK, AMP-activated protein kinase; CO, cardiac output; Dox, doxorubicin; IP, intraperitoneal; HR, heart rate; LF/HF, low frequency/high frequency; MAP, mean arterial pressure; Mel, melatonin; MKD, mg/kg/day; p, p wave interval; PR, PR interval; QRS, QRS duration; QT, QT interval; QTc, corrected QT interval; R, R wave amplitude; RR, R-R interval; ST, ST segment amplitude; STE, ST-elevation amplitude; SV, stroke volume; YAP, Yes-associated protein. Each arrow symbol represents a comparison to the control group: ↔ indicates no significant change compared to the control group. ↑, ↑↑, ↑↑↑ indicate a significant increase compared to the control group. ↑↑ indicates a further increase compared to the previous condition in the same model, which showed a ↑ compared to the control group. ↑↑↑ indicates a further increase compared to the condition in the same model, which showed a ↑↑ compared to the control group. ↓, ↓↓, ↓↓↓ indicate a significant decrease compared to the control group. ↓↓ indicates a further decrease compared to the previous condition in the same model, which showed a ↓ compared to the control group. ↓↓↓ indicates a further decrease compared to the condition in the same model, which showed a ↓↓ compared to the control group.

## Data Availability

Not applicable.
